# Epigenetic Regulation of Autophagy Beyond the Cytoplasm: A Review

**DOI:** 10.3389/fcell.2021.675599

**Published:** 2021-06-14

**Authors:** Yin Shi, Han-Ming Shen, Vidya Gopalakrishnan, Nancy Gordon

**Affiliations:** ^1^Division of Pediatrics, The University of Texas MD Anderson Cancer Center, Houston, TX, United States; ^2^Department of Biochemistry, Zhejiang University School of Medicine, Hangzhou, China; ^3^Faculty of Health Sciences, University of Macau, Macau, China

**Keywords:** autophagy, epigenetic, chromatin, histone deacetylases, methyltransferases

## Abstract

Autophagy is a highly conserved catabolic process induced under various stress conditions to protect the cell from harm and allow survival in the face of nutrient- or energy-deficient states. Regulation of autophagy is complex, as cells need to adapt to a continuously changing microenvironment. It is well recognized that the AMPK and mTOR signaling pathways are the main regulators of autophagy. However, various other signaling pathways have also been described to regulate the autophagic process. A better understanding of these complex autophagy regulatory mechanisms will allow the discovery of new potential therapeutic targets. Here, we present a brief overview of autophagy and its regulatory pathways with emphasis on the epigenetic control mechanisms.

## Introduction

Macroautophagy (hereafter called autophagy), derived from a Greek term that refers to “self-eating,” is an evolutionary conserved and precisely regulated multi-step process that involves the engulfment of organelles and proteins into a double-membrane structure called the autophagosome, followed by fusion with a lysosome for degradation. Autophagy thus recycles cellular contents to provide necessary nutrients and molecular building bricks to the cell, serving as a powerful booster of metabolic homeostasis. A growing body of evidence indicates the importance of autophagy in various physiologic and pathologic processes and its implications in human health and diseases, such as cancer, neurodegenerative disease, and the immune system (Choi et al., 2013; Baek and Kim, 2017, Amaravadi et al., 2019). However, a clear understanding of the role epigenetics plays within these processes as it relates to autophagy is not known. Here we focus on some of the known epigenetic mechanisms involved in the regulation of autophagy.

## Autophagy Regulatory Signals

The process of autophagy has been drawing increasing attention owing to its complexity and its function in the control of many different diseases and processes. Primarily, autophagy acts as a powerful booster for the cellular metabolic homeostasis, as it constitutes the main mechanism of cellular degradation triggered by nutrient deprivation and maintains not only the cellular amino acid pool, but also the recycling of other types of nutrients, such as lipids and carbohydrates (Lum et al., 2005; Onodera and Ohsumi, 2005, Lecker et al., 2006; Poillet-Perez et al., 2018). In addition, autophagy is a well-known but paradoxical determining factor in cell survival and cell death. The nutrient-recycling function of autophagy serves as a pro-survival mechanism, especially under starvation or energy-deprivation conditions (Onodera and Ohsumi, 2005; Shen and Codogno, 2011, Amaravadi et al., 2019). This pro-survival role has been validated using various animal models deficient in specific autophagy-related genes, such as autophagy-related gene 3 (*Atg3*), *Atg5*, *Atg7*, and *Atg16* (Kuma et al., 2004; Komatsu et al., 2005, Saitoh et al., 2008; Sou et al., 2008, Saitoh et al., 2009). However, under certain circumstances, autophagy is also able to mediate cell death. For instance, autophagy is reported to contribute to developmental cell death of the *Drosophila* salivary gland, midgut, and reproductive tissues (Berry and Baehrecke, 2007; Hou et al., 2008, Denton et al., 2010; Nezis et al., 2010).

Similar to other diseases and processes, autophagy and its function in cancer development remains highly debated. Autophagy has been shown to play both tumor-suppressive and promoting roles in cancer development, and accumulating evidence supports this duality (White, 2012; Amaravadi et al., 2019). In the initiation of tumorigenic and oncogenic transformation, autophagy plays a suppressive role. Depletion of several genes essential for autophagy in various mouse tissues, including *Beclin1*, *Atg5*, and *Atg7*, leads to tissue-specific tumor formation (Liang et al., 1999; Qu et al., 2003, Yue et al., 2003; Ding et al., 2008, Takamura et al., 2011), suggesting that autophagy functions as a tumor suppressor in the early stages of tumorigenesis. It is also believed that autophagy plays an important role in preventing DNA damage and maintaining genome stability, thereby suppressing tumorigenesis (Rabinowitz and White, 2010; Guo et al., 2013a). It also contributes to tumor suppression by helping reduce the harmful accumulation of reactive oxygen species and other damaged proteins (Mathew et al., 2007, 2009). For instance, deficiency of autophagy leads to p62 accumulation, induction of chronic tissue damage and inflammation, transcription of antioxidant-defense genes, and increased tumorigenesis of benign liver hepatomas (Komatsu et al., 2010; Lau et al., 2010, Takamura et al., 2011).

However, during the late stages of cancer development, autophagy is a known cancer promoter, especially in solid tumors. Under nutrient starvation or other stress conditions, such as oxidative stress or DNA damage, the pro-survival mechanism of autophagy serves to promote tumor growth (White, 2012). For instance, in Ras-driven tumors, autophagy promotes tumor cell proliferation and tumorigenesis by maintaining cellular metabolism (Guo et al., 2011, 2013b). Thus, targeting autophagy is a potential alternative anti-cancer therapy for certain tumor types. This tumor promoter role has been supported in various animal models where essential Atg genes have been depleted. For instance, depletion of *Atg5* and *Atg7* in lung cancer reduced progression from adenomas to adenocarcinomas (Strohecker et al., 2013; Rao et al., 2014). Other features of autophagy that can benefit tumor cells under harmful environmental conditions include the promotion of genome stability and cellular control of reactive oxygen species or other damaged proteins (White, 2012). Therefore, inhibition of autophagy under these conditions may be a valuable tool as anti-cancer therapy, especially when used in combination with standard therapeutic approaches.

Overall these different functions of the autophagy process rely on specific known and not well known regulatory mechanisms. Below we will give and overview of the regulatory signals that control autophagy with particular emphasis on the epigenetic control.

### mTORC1 and AMPK as the Main Regulators of the Autophagy Process

Autophagy is controlled by a group of proteins encoded by the *Atg* genes, most of which are essential for autophagy in yeast as well as in humans (Meijer et al., 2007, Rubinsztein et al., 2012; Choi et al., 2013). These genes regulate the autophagy process during: (1) the early stage, involving the formation of a complete double-membrane structure, the autophagosome and (2) the late stage, which involves the maturation, formation of the autolysosome via fusion with a lysosome and the degradation process.

The initiation/nucleation and elongation steps constitute early autophagic steps. Briefly, autophagy is initiated with the formation of a phagophore structure, controlled by the Unc-51 like autophagy activating kinase 1 (ULK1)-*Atg1* complex (Mizushima, 2010). This complex is controlled mainly by two kinases: the mammalian target of rapamycin complex 1 (mTORC1) and the adenosine monophosphate-activated protein kinase (AMPK; Hosokawa et al., 2009; Mizushima, 2010, Rabinowitz and White, 2010). mTORC1 is well-known as a key positive regulator of cell growth and protein synthesis (Jewell et al., 2013). Inhibition of mTORC1 using the specific inhibitor rapamycin or through deprivation of amino acids induces autophagy by changing the phosphorylation of ULK1, *Atg13* and other proteins in the complex including FIP200, stimulating ULK1 kinase activity, required for the initiation of autophagy (Wang et al., 2017). The AMPK signaling pathway is critical for the glucose starvation/energy deprivation response. Under such conditions, AMPK induction leads to autophagy initiation via the following pathways: (1) direct activation of the ULK1-*Atg1* complex and Beclin1 through phosphorylation and (2) suppression of mTORC1 by tuberous sclerosis complex 2 (TSC2) and Raptor phosphorylation (Gwinn et al., 2008; Shaw, 2009, Kim et al., 2011; Zhang et al., 2016). Table 1 summarizes the phosphorylation sites of autophagy related genes induced by the AMPK/mTOR signaling pathway.

**TABLE 1:** Phosphorylation sites of autophagy related genes induced by the AMPK/mTOR signaling pathway.

**Autophagy related genes**	**PTM**	**Activation dependent on**	**Function**	**References**
ULK1	Phosphorylation (Ser 317/Ser777)	AMPK	mTORC1 inhibition-autophagy induction under starvation	Kim et al., 2011
ULK1	Phosphorylation (Ser555)	AMPK	ULK1 binding to 14-3-3	Bach et al., 2011
ULK1	Phosphorylation (Ser555, 637;Thr659)	AMPK	Localization of ATG9 to perinuclear clusters	Mack et al., 2012
ULK1	Phosphorylation (Ser638,Ser758)	mTORC1	Inhibits autophagy-blocks binding of ULK1 to AMPK	Shang et al., 2011
BECN1	Phosphorylation (Ser91,Ser94)	AMPK	Enhances PtdIn3K complex activity	Fogel et al., 2013; Kim et al., 2013
ATG13	Phosphorylation (unknown)	TORC1	Blocks autophagy and interaction between ATG13 and ATG1	Kamada et al., 2010

The nucleation and elongation steps are mediated by the class III phosphatidylinositol 3-kinase (PI3K)-Beclin1 complex (Wong et al., 2011; Choi et al., 2013). The Beclin1 membrane-binding domain participates in the nucleation process (Huang et al., 2012), where the nucleated membrane structures are further elongated and completed. This step is mediated by two ubiquitin-like conjugation systems: *Atg12-Atg5* and microtubule-associated protein 1 light chain 3 (LC3)-phosphatidylethanolamine (Xie and Klionsky, 2007). These two highly conserved conjugation systems are similar in nature and are interconnected. The two systems function via attachment of small molecules to proteins through a ubiquitin-like system that involves *Atg12-Atg5-Atg16L* conjugation, which in turn facilitates the lipidation of LC3, thus promoting the attachment of LC3 to the autophagosome membrane (Xie and Klionsky, 2007).

The late stage, which includes the maturation and degradation step, involves fusion of the outer membrane of the autophagosome with the late endosome or lysosome to form the autolysosome, where the acidic lysosomal hydrolases degrade the inner membrane of the autophagosome and its luminal contents for further recycling and reuse (Choi et al., 2013).

### Nuclear Signals in Autophagy Regulation

Historically, autophagy was believed to be an exclusively cytosolic process. For instance, previous reports demonstrated the ability of enucleated cells to still form an autophagosome and maintain a complete autophagy process (Morselli et al., 2011; Feng et al., 2014). However, recent studies uncovered several nuclear events as essential for the autophagy process and outlined the importance of these events in autophagy regulation.

Transcription factors directly mediating autophagy and lysosome gene expression are now being recognized as essential nuclear regulatory events in the autophagy process (Di Malta et al., 2019). Bioinformatics analysis demonstrated that many lysosomal genes that share one or more coordinated lysosomal expression and regulation (CLEAR) motifs (GTCACGTGAC) are specifically recognized by members of the Microphthalmia family of bHLH-LZ transcription factors (MiT/TFE; Sardiello et al., 2009; Settembre et al., 2011). Activation of these transcription factors is crucial in the regulation of autophagy and lysosomal biogenesis. More specifically, the MiT/TFE member transcription factor EB (TFEB), considered a master gene for lysosomal biogenesis, was reported to promote the expression of many genes involved in different autophagy steps, including *BECN1*, WD repeat domain phosphoinositide-interacting protein 1 (*WIPI1*), and *Atg9B*, involved in the initiation step, as well as LC3B, gamma-aminobutyric acid receptor-associated protein (*GABARAP*), and *Atg5*, involved in the autophagosome maturation and elongation step (Sardiello et al., 2009; Palmieri et al., 2011, Settembre et al., 2011; Martina et al., 2014). The role of MiT/TFE members in the regulation of autophagy and lysosome biogenesis is largely dependent on mTORC1 activity, which also coordinates the cytoplasmic autophagic signaling process. mTORC1 directly phosphorylates TFEB, and the phosphorylated TFEB is retained in the cytosol via binding to protein 14-3-3, whereas under nutrient deprivation conditions and/or mTORC1 inhibition, TFEB and various other MiT/TFE members are dephosphorylated and translocated into the nucleus, leading to lysosomal biogenesis and autophagy induction (Peña-Llopis et al., 2011; Settembre et al., 2012).

Additional nuclear transcription factors that play a role in autophagy regulation, include Forkhead box O proteins (FOXOs), p53, cAMP response element-binding protein farnesoid X receptor (CREB-FXR), and sterol regulatory element binding transcription factor 2 (SREBP2; Seo et al., 2011; Calkin and Tontonoz, 2012, Webb and Brunet, 2014). Like TFEB, these transcription factors induce the expression of several genes responsible for autophagy induction and can be directly controlled by similar upstream signaling molecules involved in the regulation of cytoplasmic autophagy, such as AKT, mTORC1, AMPK, and PI3K (Seo et al., 2011; Calkin and Tontonoz, 2012, Webb and Brunet, 2014).

Furthermore, many proteins important for the cytoplasmic autophagic signaling process were, surprisingly found to also locate into the nucleus and play a role in the regulation of autophagy. These proteins include LC3, glyceraldehyde 3-phosphate dehydrogenase (GAPDH), and VPS34 (Chang et al., 2015; Huang et al., 2015, Su et al., 2017). The translocation of these proteins is highly dependent on environmental signals that directly affect autophagic responses. For example, for decades, LC3 has been recognized as a cytoplasmic protein that functions as a marker of autophagosome formation. However, recent reports clearly show its nuclear location under normal/basal autophagy. Within the nucleus, LC3 maintains a highly acetylated state. Upon starvation, LC3 is deacetylated by Sirtuin 1 (SIRT1) and translocated into the cytoplasm, where it interacts with *Atg7*, leading to autophagosome formation (Huang et al., 2015). Lastly, nuclear regulation of autophagy is linked to epigenetic regulation, which ties transcription factors with autophagic proteins, enabling more precise complex regulation of the autophagy process (Bhol et al., 2019).

### Epigenetic Factors in Autophagy Regulatory Signaling

Genetic information contained in the DNA is shared by every cell in the body. Epigenetics determines how the genome is read and transcribed in response to different environmental signals by controlling the chromatin structure and regulating its accessibility to gene transcription. Epigenetic modifications include modifications of both DNA and histones, such as methylation and acetylation (Sharma et al., 2010; Dawson and Kouzarides, 2012).

DNA methylation is a stable gene-silencing mechanism catalyzed by DNA methyltransferases (Dawson and Kouzarides, 2012; Smith and Meissner, 2013). DNA methyltransferases can methylate DNA cytosine residues to 5-methylcytosine (5mC), while ten-eleven translocation (TET) family members demethylate 5mC residues by oxidation and subsequent loss of the methyl group (Tahiliani et al., 2009). Histone proteins can also be post-translationally modified via acetylation, methylation, phosphorylation, ubiquitylation, SUMOylation, glycosylation and ADP-ribosylation (Peterson and Laniel, 2004). Histone acetyltransferases (HATs) acetylate histone proteins, and histone deacetylases (HDACs) deacetylate them (You and Jones, 2012). The histone tail and the nucleosomal DNA are tightly associated and maintain a positively charged compact chromatin. Acetylation at lysine residues on histone tails neutralizes the positively charged chromatin, decreases the association, opens it up, and allows gene transcription (You and Jones, 2012; Roberti et al., 2019). In contrast, the function of histone methylation is dependent upon different regulatory signals and the gene expression status. Histone methylation occurs by the addition of a methyl group to the side-chain nitrogen atoms of both lysine and arginine residues. Methylation causes different transcription outcomes depending upon changes in chromatin structure, transcription factor recruitment, and association with initiation and elongation factors (Roberti et al., 2019). How some of these processes interconnect with autophagy will be reviewed in the following sections.

Table 2 summarizes the epigenetic factors known to play a role in autophagy regulation.

**TABLE 2:** Epigenetic factors involved in autophagy signaling.

**Epigenetic modification**	**Epigenetic factor**	**Modification site**	**Effect on autophagy**	**Transcription factors involved**	**References**
**Involved in AMPK signaling**					
Histone acetylation	SIRT1	H4K16	+	FOXO1, TFEB	Cantó et al., 2009; Chang et al., 2015; Sakamaki et al., 2017
	BRD4		–		Sakamaki et al., 2017
	HDAC4/5	H4K16	–		McGee et al., 2008; Chen et al., 2015
	p300		–		Yang et al., 2001; Zhang et al., 2011
	HAT1	H5K5/K12	+		Marin et al., 2017
Histone methylation	EZH2	H3K27	–		Wan et al., 2018
	G9a	H3K9	–		Artal-Martinez de Narvajas et al., 2013; Li et al., 2015; Park et al., 2016; An et al., 2017; Sakamaki et al., 2017
	KDM2A	H3K9	–		Tanaka et al., 2015; Wang et al., 2017
	CARM1	H3Arg17	+	TFEB	Shin et al., 2016
DNA methylation	DNMT1	H3K27	+		Marin et al., 2017; Yang et al., 2018
Histone phosphorylation	H2B	Ser36	+		Bungard et al., 2010; Marin et al., 2015
**Involved in mTORC signaling**					
Histone acetylation	GCN5	K274/K279	+	TFEB	Laboucarié et al., 2017; Wang et al., 2020
	p300		–		Wan et al., 2017
	hMOF	H4K16	–		Fullgrabe et al., 2013
Histone methylation	EZH2	H3K27	–		Wei et al., 2015

*+, promotes autophagy; –, inhibits autophagy.*

#### Epigenetic Factors and AMPK Signaling

**FIGURE 1:**
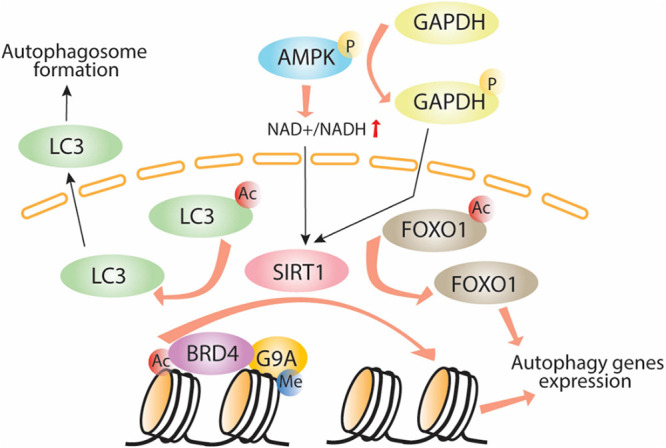
AMPK-SIRT1-autophagy signaling pathway. Adenosine monophosphate phosphorylates GAPDH which in turn translocates to the nuclei, activates Sirt1 through direct interaction allowing LC3 and histone deacetylation, Brd4 release, recruitment of the methyltransferase G9a and induction of autophagy. Ac, acetylation; P, phosphorylation; Me, methylation.

Adenosine monophosphate-activated protein kinase plays a major role in bioenergetics and energy balance by inducing direct phosphorylation of metabolic enzymes and nutrient transporters such as adipose triglyceride lipase (ATGL), glucose transporter 1 (GLUT1), and acetyl-CoA carboxylase 1 (ACC1; Fullerton et al., 2013; Herzig and Shaw, 2018). AMPK also acts as a key regulator of epigenetic events by direct phosphorylation of histones, DNA methyltransferases, and histone post-translational modifiers (Bungard et al., 2010; Marin et al., 2017, Wan et al., 2018). It influences HAT and HDAC function through phosphorylation of their cofactors or by interfering with substrate availability. Activation of AMPK increases the nicotinamide adenine dinucleotide (NAD+):NADH ratio, thus increasing the activity of one of the class III HDACs, SIRT1, which induces autophagy by enhancing deacetylation of *Atgs* and FOXO1 (Cantó et al., 2009). Another mechanism of AMPK-mediated SIRT1 activation occurs through GAPDH (Figure 1). When phosphorylated by AMPK, GAPDH translocates to the nucleus and interacts with SIRT1, which releases SIRT1 from its repressor and activates its function (Chang et al., 2015). Despite the function of SIRT1 in deacetylation of LC3 and induction of autophagy, AMPK-mediated SIRT1 activation leads to histone deacetylation and release of the epigenetic acetylation reader bromodomain-containing protein 4 (BRD4) from the promoter regions of autophagy and lysosomal genes leading to transcriptional activation of autophagy. This process occurs under nutrient deprived conditions. Under nutrient rich conditions, BRD4 binds to promoter regions of autophagy and lysosomal-related genes recruits the methyltransferase G9a, which in turn represses the transcriptional program by histone demethylation leading to suppression of autophagy and lysosomal gene expression (Sakamaki et al., 2017), thus demonstrating the importance of the BRD4/G9a interaction in the induction/repression of autophagy (Artal-Martinez de Narvajas et al., 2013; Li et al., 2015, Park et al., 2016; An et al., 2017).

Several reports also indicate that AMPK directly phosphorylates two class IIa HDACs, HDAC4 and HDAC5, increasing their cytoplasmic translocation and interaction with 14-3-3 proteins (McGee et al., 2008; Chen et al., 2015). HDAC4 and HDAC5 lost interaction with 14-3-3 proteins leads to their nuclear translocation and further interaction with HDAC 3 leads to repression of gene expression. Thus HDAC 4 and HDAC5 when localized to the nucleus can function to inhibit autophagy. Inhibition of HDAC 5 have been shown to induce autophagy (Peixoto et al., 2012). HDAC 6 independently of AMPK has been shown to control autophagolysosome fusion. HDAC 6 deficiency leads to failure of autophagosome maturation and build up of protein aggregates (Lee et al., 2010).

Adenosine monophosphate-activated protein kinase environmental conditions determine its role in the HAT activity. For example, p300 HAT functions as a histone acetyltransferase that regulates transcription via chromatin remodeling (Lee and Finkel, 2009). p300 phosphorylation by AMPK leads to acetylation and transcriptional activation of target genes some of them components of the autophagy machinery (Yang et al., 2001; He et al., 2009, Lee and Finkel, 2009; Zhang et al., 2011, Lim et al., 2012). Further studies also demonstrated that HAT1 contains the AMPK consensus phosphorylation sequence, hence HAT1 function could be promoted by AMPK, which acetylates histones to favor transcription of genes related to mitochondrial biogenesis (Marin et al., 2017). In addition, AMPK can regulate acetylation by modulating availability of acetyl-CoA, the main acetyl donating group of acetylation. Acetyl-CoA level can be changed (1) by AMPK activation through its direct phosphorylation of ACC1, which prevents its role in the conversion of acetyl-CoA to malonyl-CoA and thus increases the acetyl-CoA level (Fullerton et al., 2013) and (2) through the acetyl-CoA synthetase short-chain family member 2 (ACSS2), also a substrate of AMPK, which converts acetate to acetyl-CoA (Li et al., 2017). When phosphorylated by AMPK, ACSS2 translocates to the nucleus and interacts with TFEB, producing acetyl-CoA locally for histone H3 acetylation, leading to induction of autophagy and lysosomal gene biogenesis (Bulusu et al., 2017; Li et al., 2017, Zhang et al., 2018).

**FIGURE 2:**
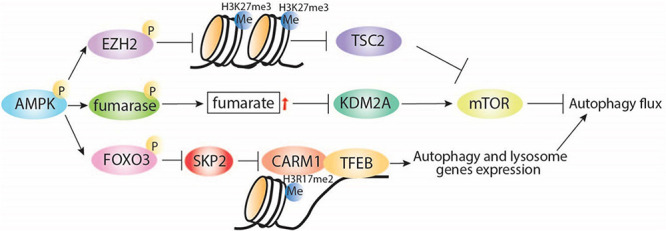
AMPK regulates autophagy through interaction with regulators of histone. AMPK inhibits H3K27me3 via direct phosphorylation of EZH2, which was reported to release TSC2 expression and then induce autophagy via inhibition of mTORC1. Under starvation conditions, AMPK can indirectly increase fumarate levels, leading to KDM2A repression, mTOR inhibition, and induction of autophagy. In addition, starvation-induced AMPK can regulate histone methylation through induction of autophagy via FOXO3 phosphorylation, transcriptional repression of SKP2, and nuclear translocation of CARM1, which directly interacts with TFEB, leading to induction of autophagy and lysosomal gene expression. P, phosphorylation; Me, methylation.

Adenosine monophosphate-activated protein kinase also plays direct and indirect roles in histone methylation. Directly, AMPK negatively regulates gene silencing by phosphorylating the histone methyltransferase enhancer of zeste homolog 2 (EZH2) and disrupting the polycomb repressive complex 2 (PRC2) mediating methylation on H3 at Lys27 (H3K27me3; Wan et al., 2018). Interestingly, EZH2-induced increase of H3K27me3 is known to repress the expression of TSC2, subsequently activating mTORC1 and inhibiting autophagy (Wei et al., 2015). Indirectly, AMPK increases the cellular fumarate level by phosphorylation and inhibition of the enzyme responsible in the conversion of fumarate to malate (Figure 2). An increase in fumarate leads to inhibition of lysine-specific demethylase 2A (KDM2A) and generation of H3K36me2 (Tanaka et al., 2015; Wang et al., 2017). KDM2A deficiency is reported to suppress mTOR activity via PI3K/AKT pathway (Lu et al., 2019), which indicates a potential indirect effect of the AMPK-KDM2A-mTOR signaling pathway on autophagy regulation (Lu et al., 2019).

Additionally, AMPK can regulate histone methylation by AMPK-mediated autophagy induction under nutrient starvation conditions (Figure 2). Starvation-induced activation of AMPK leads to FOXO3a phosphorylation and transcriptional repression of S-phase kinase-associated protein 2 (SKP2) expression. Decreased SKP2 levels reduce SKP2-containing SCF (SKP1–cullin1–F-box protein) E3 ubiquitin ligase and nuclear translocation of coactivator-associated arginine methyltransferase 1 (CARM1) (Shin et al., 2016). CARM1 is essential for autophagy in mammals, promoting TFEB-mediated induction of autophagy and lysosome gene expression. These observations highlight the interconnection between energy sensing and transcriptional and epigenetic regulation of autophagy.

Adenosine monophosphate-activated protein kinase has also been shown to play a role in DNA methylation and hence, indirectly, in autophagy regulation. For instance, DNA methyltransferase 1 (DNMT1) is a phosphorylation substrate of AMPK; AMPK-mediated DNMT1 phosphorylation inhibits its function and thus reduces DNA methylation, enhances the accessibility of DNA to promoters, and induces mitochondrial gene expression (Marin et al., 2017). More specifically, DNMT1 induces DNA methylation on the cystic fibrosis transmembrane conductance regulator (CFTR) promoter and inhibits its expression. CFTR is known to enhance autophagosome formation via induction of Beclin1, LC3, and Atg12 expression (Yang et al., 2018).

Adenosine monophosphate-activated protein kinase’s effect on histone phosphorylation has also been linked to regulation of autophagy. Bioinformatics analysis has recently demonstrated the AMPK phosphorylation consensus sequence to be found in various histone proteins (Marin et al., 2015). Experimental proof of the histone phosphorylation by AMPK has also been reported. Bungard et al. found that activation of AMPK by glucose starvation or ultraviolet radiation leads to its direct translocation to the chromatin and phosphorylation of histone H2B at serine 36 (Bungard et al., 2010). H2B serine 36 phosphorylation is essential for cell survival in response to glucose- or energy-limited conditions. Phosphorylation of H2B was also shown by Liu et al. to increase autophagy in colon cancer cells via enhancement of *Atg* genes transcription (Liu et al., 2020), which might indicate an additional indirect effect of AMPK signaling on autophagy induction.

#### Epigenetic Factors and mTOR Signaling

Mammalian target of rapamycin protein controls the translation process in response to nutrient stress signals; under nutrient-limited conditions, mTOR is inhibited and autophagy is induced. Recent studies link mTOR/TOR to the histone acetyltransferase GCN5 and to nutrient response (Laboucarié et al., 2017; Wang et al., 2020). In fission yeast, starvation-mediated TORC1–protein phosphatase 2A (PP2A) signaling and TORC2-AKT signaling induced phosphorylation of a key component of the Spt-Ada-Gcn5 acetyltransferase (SAGA) complex, transcription initiation factor TFIID subunit 12 (Taf12). In turn, activation of SAGA downstream of TORC1 led to yeast response to nutrient starvation (Laboucarié et al., 2017). Consistently, in mammalian and *Drosophila* models, induction of autophagy led to inhibition of the histone acetyltransferase GCN5, which led to decreased TFEB acetylation and increased lysosome and autophagic gene expression (Wang et al., 2020). Further evidence suggests that mTORC1 inhibition by Torin1 or amino acid–starvation treatments leads to decreased GCN5 activity and inhibition of autophagy (Wang et al., 2020). GCN5 is also known to inhibit autophagy through direct acetylation of Atg7 in yeast (Zhang et al., 2017). Perhaps, the mTOR/TOR regulatory effect on GCN5 and its downstream acetylation targets highlights the potential relevance of this pathway in the epigenetic regulation of autophagy and lysosome gene expression.

**FIGURE 3:**
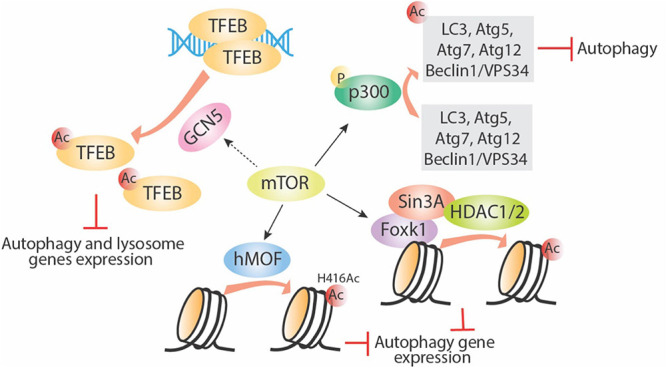
mTOR regulates autophagy via targeting acetyltransferases. mTOR can enhance activity of acetyltransferases including p300, GCN5, and hMOF. Phosphorylation of p300 directly acetylates several important autophagy-related proteins and inhibits their function; GNC5 acetylates TFEB and inhibits its downstream autophagy and lysosome gene expression; hMOF acetylates histone proteins directly and inhibits autophagy gene expression; mTOR also increase Foxk1 nuclear location, then the Foxk1 recruits Sin3A-HDAC1/2 complex to chromatin and subsequently decreases autophagy gene expression. Ac, acetylation; P, phosphorylation.

As shown in Figure 3, several other acetyltransferases are involved in mediating mTOR-regulated autophagy. An important link established between mTOR and p300 HAT has revealed the pivotal role of mTOR in cell metabolism through regulation of autophagy and lipogenesis (Wan et al., 2017). p300 inhibits autophagy through acetylation of some important autophagy proteins, including LC3, Beclin1/VPS34, Atg5, Atg7, and Atg12 (Lee and Finkel, 2009; Sun et al., 2015, Su et al., 2017). mTOR can phosphorylate and activate p300 directly, thereby inhibiting autophagy (Wan et al., 2017). Furthermore, the promotion of autophagy through rapamycin-mediated inhibition of mTORC1 activity has been associated with a reduction in histone H4 lysine 16 acetylation (H4K16ac) and the human ortholog of *Drosophila* males absent on the first (hMOF). Rapamycin-induced mTOR inhibition enhances deacetylation of H4K16 and decreases Atg gene expression and this effect was dependent upon a balance between hMOF and SIRT1 (Fullgrabe et al., 2013). This effect can be bypassed by the addition of the HDAC inhibitor valproic acid, which in the presence of rapamycin increases H4K16 acetylation and induction of autophagic flux. Thus, a novel negative feedback network exists between mTOR, hMOF, H4K16ac, and autophagy outcome (Fullgrabe et al., 2013). Aside from the effect of mTORC1 on specific histone acetylation enzymes, Shi et al. recently showed that mTORC1 can also directly affect fatty acid synthesis, a major source of acetyl-CoA for histone acetylation, thus causing an additional selective effect on gene expression specifically in dendritic cells (Shi et al., 2019).

Not much is known about mTORC1 and histone methylation. However, there is some evidence of a relationship between mTORC1, histone methylation, and regulation of autophagy. Starvation-induced mTORC1 inhibition and autophagy induction is linked to a decrease in H3K4me2 and an increase in H3K4me3 (Lee et al., 2017). Studies in yeast showed the importance of TORC1 and histone methylation during the nutrient stress response (McDaniel et al., 2017). In that study, H3K36 methylation and its specific methyltransferase, SET domain containing 2 (Set2), are required for the transcriptional response to nutrient stress. Set2 has been found to interact with TORC1 and TORC2. Although the mechanism is unclear, TORC1 signaling appears to be disrupted in Set2-deficient cells, suggesting a potential role of Set2-H3K36 methylation in TORC1 regulation (McDaniel et al., 2017). In addition, mTOR is also reported to limit basal autophagy via the well-conserved mTOR-Foxk1-Sin30HDAC1/2 axis. Under nutrient full conditions, mTOR enhances the nuclear entry of Foxk1, which recruits the Sin3-HDAC1/2 complexes to reduce the acetylation of H4 and essential autophagy genes expression (Bowman et al., 2014). Lastly, in mammalians, the key methyltransferase EZH2, which mainly causes trimethylation of H3K27, was found to silence a series of negative regulators of mTORC1, especially the TSC2, leading to activation of mTORC1 and subsequent inhibition of autophagy (Wei et al., 2015).

In summary, we provide an overview of various published concepts that suggest a potential relationship between epigenetic modifications and the mTOR signaling pathway. Further studies are needed to better understand this relationship.

#### Epigenetic Factors Involved in Other Autophagy-Regulatory Pathways

Alongside the mTOR-related and AMPK-related epigenetic processes that play a role in the regulation of autophagy, substantial evidence demonstrates the involvement of several other epigenetic factors that modulate autophagy and various other downstream metabolic events. For instance, under starvation conditions, the deubiquitinase USP44 decreases H2B monoubiquitination (H2Bub1), decreasing H4K16ac and the activity of its acetyltransferase hMOF, which in turn changes the transcription of various autophagy-regulatory genes to initiate autophagy (Chen et al., 2017).

Interestingly, reducing expression of some Atg genes by methylation of their promoter regions down-regulates autophagy directly, and these epigenetic reprogramming occurs in tumor or aging cells. For instance, hyper-methylation of Beclin1 promoter regions has been found in breast tumors cells (Li et al., 2010). In childhood Acute Lymphatic Leukemia derived cells, expression of LC3B and Atg5 was found to be reduced due to methylation on the promoter regions (Hassen et al., 2017). Lastly, in macrophages from aged mice, the activity of DNA methyltransferase 2 (DNMT2) appeared to be increased due to methylation of Atg5 and LC3 promoter regions (Khalil et al., 2016).

Most recently, the histone demethylase Jumonji-D3 (JMJD3/KDM6B) was found to mediate Fibroblast Growth Factor-21 (FGF21) induced autophagy and lipid degradation through a mechanism that involves activation of protein kinase A (PKA) and subsequent phosphorylation and activation of JMJD3. Active JMJD3 demethylates histone H3K27-me3, which leads to global autophagy genes expression (*Tfeb, Atg7, LC3*, and *Ulk1*) (Byun et al., 2020).

Additionally, the histone demethylase lysine-specific demethylase 1 (LSD1) is reported to be involved in the regulation of autophagy, in particular the autophagic degradation of intracellular lipid droplets, known as lipophagy. LSD1 is recruited to TFEB by the small heterodimer partner (SHP), a key transcriptional regulator responsible for maintaining bile acid homeostasis and responses to a late fed-state hormone. LSD1 recruitment causes demethylation of H3K4me2/3, leading to suppression of gene transcription. Under these conditions, activation of the FGF19-SHP-LSD1 pathway triggers a nutrient-rich postprandial signal response to inhibit autophagy, particularly lipophagy in liver tissue (Byun et al., 2017). LSD1 has also been shown to decrease p62—also termed sequestesome 1(SQSTM1)—protein stability in a demethylation-independent manner and inhibit autophagy in gynecologic malignancies. Combination LSD1 inhibitor and autophagy blockade decreases tumor growth (Chao et al., 2017).

Finally, histone activation of the epigenetic marks H3K4me3, H3K27ac, and H3K56ac increases transcription of autophagy-related genes under nutrient-deprived conditions (Peeters et al., 2019). Supporting this finding is a study demonstrating that, in autophagy induced by Epstein-Barr virus nuclear antigen 3C (EBNA3C), EBNA3C can recruit several HATs and HDACs and disrupt various histone modifications, leading to activation of some histone epigenetic marks, including H3K4me1, H3K4me4, H3K9ac, and H3K27ac, and transcriptional activation of Atg genes, such as *Atg3*, *Atg5*, and *Atg7* (Bhattacharjee et al., 2018). Overall, these studies provide direct evidence of the global epigenetic changes that influence autophagy induction. Further understanding of the mechanisms underlying these epigenetic changes might provide meaningful information on potential novel epigenetic targets that can be used to modulate the autophagy process.

## Conclusion

The two master regulators of autophagy, AMPK and mTORC1, are the main sensors of the cellular environmental changes that determine an autophagy response. Epigenetic modifications play a crucial role in the regulation of gene expression. Despite the evolving significance given to the transcriptional regulation of autophagy, the role for epigenetic control is mostly unknown. Here we give an overview of the epigenetic events that influence the autophagy process by altering the activity of transcription factors that lead to changes in autophagy and lysosomal related gene expression. Since epigenetic processes are reversible, a better understanding of the link between epigenetics and autophagy might offer therapeutic opportunities.

## Author Contributions

YS and NG contributed to the conception and design of the review. YS created the figures and tables. H-MS and VG wrote sections of the review. H-MS and NG edited the review to its final version. All authors contributed, agreed, and approved the submitted version of the manuscript.

## Conflict of Interest

The authors declare that the research was conducted in the absence of any commercial or financial relationships that could be construed as a potential conflict of interest.
